# A pilot study for the isolation of *Eimeria* spp. oocysts from environmental straw samples in comparison with individual faecal examination of fattening calves

**DOI:** 10.1007/s00436-023-07876-6

**Published:** 2023-05-31

**Authors:** Jessica Bauer, Martin Kaske, Andreas Oehm, Manuela Schnyder

**Affiliations:** 1 Institute of Parasitology, Vetsuisse Faculty, University of Zurich, Winterthurerstrasse 266 A, 8057 Zurich, Switzerland; 2 Swiss Calf Health Service, University of Zurich, Winterthurerstrasse 260, 8057 Zurich, Switzerland

**Keywords:** *Eimeria* oocysts, Calves, Environmental contamination, Straw, Quantitative oocyst isolation technique

## Abstract

The diagnosis of eimeriosis in calves mainly relies on the presence of diarrhoea and the excretion of *Eimeria* oocysts in the faeces. Restraining the animals to collect rectal samples for diagnostic purposes is stressful and time-consuming. The aim of this study was to evaluate a method for the quantification of oocysts in environmental barn straw samples. To investigate the recovery rate of the method, straw and *Eimeria* negative faeces were spiked with *Eimeria* oocysts in plastic bags and mixed with water and 0.05% Tween 20 (v/v); the liquids were filtered twice through sieves (mesh size 300 and 52 μm), centrifuged and the number of oocysts in the sediment determined using a McMaster counting chamber. A recovery rate of 52.4% (95% confidence interval: 48.2–56.5%) was obtained. In the following, field straw (*n* = 156) and individual faecal samples (*n* = 195, also analysed by McMaster counting chambers) were collected on four different farms. *Eimeria* oocysts were present on all farms in faecal (84/195, 43.1%) and straw samples (119/156, 76.3%). In 37 (23.7%) straw samples, sporulated oocysts were observed, with a sporulation rate ranging from 0 to 40%. Despite high variability between farms and examination days, mean numbers of oocysts in the straw positively correlated with mean numbers of oocysts excreted in the faeces (*ρ*_*Spearman*_ = 0.60). The examination of environmental straw samples may represent an easy-to-perform, non-invasive, inexpensive preliminary diagnostic approach for surveillance of eimeriosis at group level, having the potential to assess the infection pressure.

## Introduction

*Eimeria* spp. are parasitic organisms known to cause intestinal lesions in calves aged 3 weeks to 12 months (Keeton and Navarre 2018); infections may entail high economic losses due to impaired performance, higher mortality, and increased costs for veterinary treatments (Fitzgerald 1980; Lassen and Ostergaard 2012; Tomczuk et al. 2015). Of the 13 species of *Eimeria* infecting cattle, *E. zuernii*, *E. bovis* and *E. alabamensis* are the most pathogenic (Svensson et al. 1994; Bangoura and Daugschies 2007; Daugschies et al. 1997; Daugschies and Najdrowski 2005; Hooshmand-Rad et al. 1994). Under conventional farming conditions, cattle are frequently harbouring *Eimeria* spp., and the importance of subclinical infections should not be underestimated (Daugschies and Najdrowski 2005). The prevalence of *Eimeria* infections in young cattle and, accordingly, the environmental contamination with oocysts can be high (Cornelissen et al. 1995; Bangoura et al. 2011; Forslid et al. 2015; Koutny et al. 2012). Different factors may have an impact on the outcome of an infection: the involved *Eimeria* species and infection dose, the immunological status and age of the host, environmental and climatic conditions and others (Lassen et al. 2009a; Niilo 1969; Taubert et al. 2008; Lassen et al. 2014). The continuous exposure to low numbers of oocysts results in endemic stability (Conlogue et al. 1984; Daugschies and Najdrowski 2005).

Diagnosis of coccidiosis is based on clinical observations and the examination of faecal samples. Quantitative determination and a species-specific differentiation of the *Eimeria* spp. are relevant, as the number of excreted *Eimeria* oocysts varies considerably depending on the time of infection (Niilo 1969; Bangoura and Daugschies 2007) and due to the presence of less pathogenic and pathogenic species (Keeton and Navarre 2018). However, rectal sample collection is stressful for the animals and laborious for farmers and practitioners. Therefore, a method to identify and quantify the infection pressure within the group without animal handling could represent an alternative.

The goal of the study was to evaluate an easy-to-apply method with high recovery rates and reproducibility for the quantification of *Eimeria* oocysts from environmental straw samples collected from calf barns, and to compare the results with numbers obtained from individual faecal samples.

## Material and methods

### Study population and faecal sampling

Four fattening beef farms were selected by the Swiss health service for calves (KGD; www.kgd-ssv.ch), based on farmer motivation to participate. During the study, no anticoccidial group treatment was performed, and no antimicrobials that could have affected *Eimeria* infections (e.g., sulfonamides) were used. Single animals were excluded from the study in case of death (one animal on farm 1 and 4 each) or if faeces could not be collected on study days. Individual animals (not excluded from the study) were treated with diclazuril (1 mg/kg body weight, Vecoxan®, oral suspension) if they showed the following clinical signs: diarrhoea (partially containing blood), inappetence, lethargy, signs of abdominal discomfort and tenesmus and if they had a high faecal oocyst excretion with a high proportion of pathogenic species (*Eimeria zuernii* and/or *E. bovis*).

All farms worked with an all-in-all-out system and rehoused calves from different farms of origin until maturity for slaughter. The calves were housed in indoor groups on deep straw barns and fed with milk replacer. In addition, calves had ad libitum access to water and hay. The farms were visited repeatedly (2–4 times each) at different time points between October 2021 and April 2022 in the period between calf arrival (day 0) up to 47 days later. The number of animals per pen ranged from 12 to 40 calves and the number of included animals per farm was 13, 18, 19 and 11 for farms 1–4, respectively. Individual faecal samples were taken rectally from each calf. Overall, 61 crossbreed calves, aged 22 to 87 days (mean: 49.5; standard deviation: 16.8), were sampled. Upon arrival at the laboratory, the samples were stored at 4 °C and analysed within 1 day.

### Analysis of faecal samples

The McMaster method (sensitivity: 50 oocysts) was applied to determine the number of *Eimeria* oocysts per gram of faeces (OPG), as previously described (Deplazes et al. 2020).

### Eimeria oocyst recovery from straw samples

#### Preparation of an Eimeria oocyst stock suspension

Non-sporulated *Eimeria* spp. oocysts were isolated from positive faecal samples of naturally infected calves with an OPG > 5000. Ten grams of faeces were mixed with tap water, filtered through a metal sieve (mesh size 300 μm) followed by a filter with mesh size 52 μm into a 250-ml beaker glass. The filters were flushed with tap water and the suspension sedimented for 2 h. The supernatant was decanted; 1–2 ml of the sediment was transferred into a 14-ml test tube and filled up with saturated sodium chloride (density 1.20 g/ml). After centrifugation at 500 g for 3 min, the supernatant was filtered through a 7-μm nylon filter. To recover the *Eimeria* oocysts, 5 ml of tap water was poured onto the filter, the liquid collected again with a pipette, transferred into a 14-ml test tube and centrifuged for 3 min at 500 g. The supernatant was discarded, and the sediments were pooled as an *Eimeria* oocyst stock suspension and stored in water at 4 °C. The concentration of the *Eimeria* oocyst stock suspension was determined using eight grids of a Neubauer chamber, and the mean was calculated to estimate the number of oocysts per millilitre. Working suspensions of the required number of *Eimeria* oocysts were prepared accordingly.

#### Evaluation of the Eimeria oocyst recovery rate from spiked straw samples

**Fig. 1:**
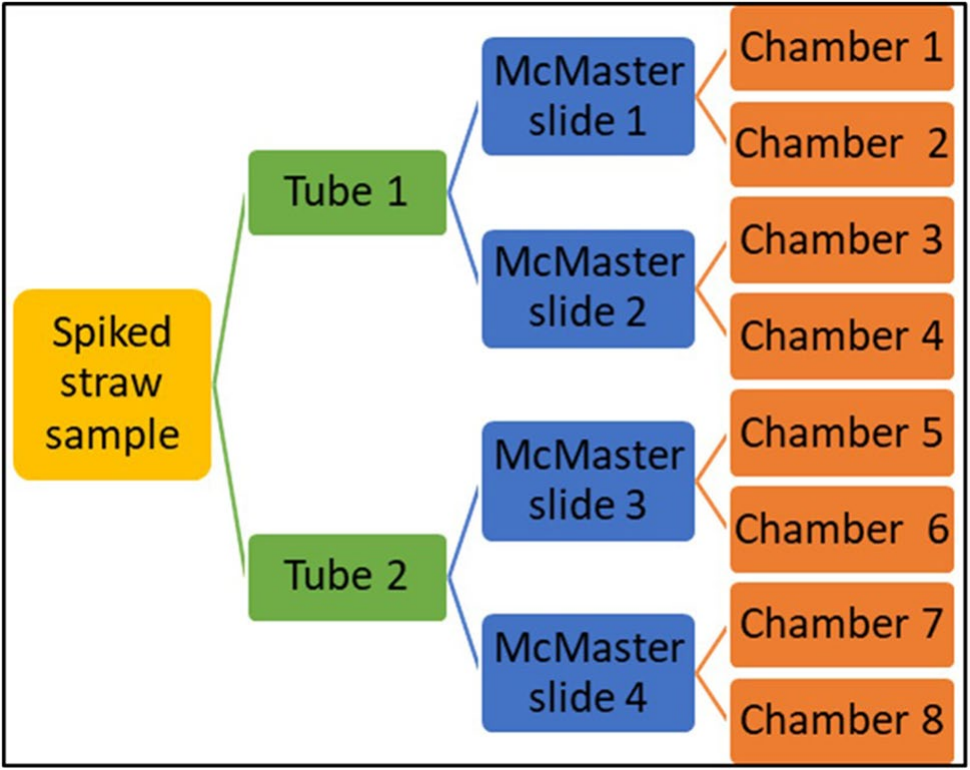
Experimental design for the evaluation of straw samples spiked with *Eimeria* oocysts. The filtered liquid was divided into two 50-ml tubes, and out of each tube, 2 McMaster slides were analysed, having each 2 chambers that were both counted out

Overall, 30 analyses were performed, i.e., 10 for each oocyst concentration 1000, 10,000 and 30,000 oocysts per straw sample. For this, straw and *Eimeria* negative faecal samples were autoclaved (121 °C, 50 min, 2 bar). Subsequently, 5 g straw, 2 g of the negative faecal sample and 2 ml tap water were put together into a plastic bag (volume: 2 l). One millilitre of the working suspension with the required number of *Eimeria* oocysts was uniformly dispersed over the straw. After adding 100 ml of tap water with 0.05% Tween 20 (v/v), the bag content was thoroughly mixed and left for 5 min. Then, one corner of the plastic bag was cut (around 5 mm), and the liquid poured through a metal sieve (mesh size 300 μm) and a nylon filter with mesh size 52 μm, into a 250-ml beaker glass. To avoid blockage with dirt, single-use plastic pipettes were used to facilitate the flow of the liquid on the 52-μm filter. The straw in the bag was pressed out manually until no further liquid could be produced. Once all the liquid had passed through, the 52-μm filter was flushed with tap water containing 0.05% Tween 20 until 100 ml was reached in the beaker glass. While the obtained suspension was on a magnetic stirrer, 50 ml was collected with a pipette and transferred into a 50-ml plastic tube. The remaining suspension was transferred into another 50-ml plastic tube. Both were then centrifuged for 3 min at 800 g. The sediments of both 50 ml tubes of each spiked straw sample were analysed using 2 McMaster slides (with 2 chambers each) for each tube (Fig. 1). For this, the supernatants were removed with a vacuum pump. Saturated sodium chloride was filled up to 30 ml and the tubes vortexed carefully, before filling the McMaster counting chambers with a pipette and counting the *Eimeria* oocysts under the microscope (100 × magnification). The number of oocysts per straw sample was therefore calculated as follows:

$$Number of oocysts=\frac{N \times FV}{VMcM } \times 2= \frac{N \times 30 \mathrm{ml}}{0.3 \mathrm{ml}} \times 2=N \times 200$$

where *N* = number of oocysts counted in both chambers of a McMaster slide, FV = final volume of the suspension containing the oocysts, and VMcM = volume of the two McMaster chambers (multiplying by 2 is based on the suspension split into two tubes, Fig. 1). The detection limit was therefore 200 oocysts per straw sample.

#### Collection of field straw samples and detection of Eimeria oocysts

The animals were kept on deep straw, with fresh straw put on top daily and without straw removal. Straw samples were always taken shortly before fresh straw was added. Based on comparable regulatory requirements of minimum surfaces for calf rearing but independently from the barn size, twelve straw samples were collected in each stable and examination day, contemporaneously with the individual faecal samples of the calves. For randomisation and to collect oocysts throughout the barns, the areas were crossed in a zigzag pattern starting from a corner, derived from studies collecting grass samples for the determination of larvae of gastrointestinal nematodes (Taylor 1939; Verschave et al. 2015). At the 12 sampling spots, a fistful of straw was collected from the surface and put into separate plastic bags (volume: 2 l). Upon arrival at the laboratory, the bags were stored at 4 °C to prevent further sporulation of the oocysts and analysed within 2 days. Ten grams of each sample were weighed, transferred into a new plastic bag, and mixed with 100 ml of tap water with 0,05% Tween 20 (v/v) and left for 5 min. Further examination steps were performed as described for the spiked straw samples, except that only one of the two 50-ml tubes was analysed for oocyst counting. The oocyst numbers of the two chambers were added up and multiplied by 200 to get the number of oocysts per straw sample. The sediment of the second 50-ml tube was split in two 14-ml test tubes, saturated sodium chloride added, and the tubes centrifuged 3 min at 500 g. Floating oocysts were then transferred to a slide using a wire loop, and from each sample, up to 50 oocysts were microscopically (400 × magnification) checked for sporulation. Only intact oocysts were examined. No *Eimeria* oocyst differentiation was performed.

Due to the different moisture content of the samples and to adequately compare the samples, the number of oocysts per gram of dry matter was determined. For this purpose, the plastic bags with the straw samples were put into a drying oven for 24 h at 70 °C and then weighed.

### Data analysis

All analyses were carried out in Microsoft Excel and the R software for statistical computing version 4.2.0 using the R Studio interface. To analyse the recovery rate, the median of the four counted McMaster slides was calculated for each spiked straw sample. The Shapiro–Wilk test was implemented to check all variables for normal distribution. A *p* value of < 0.05 implied that the distribution of the variables ‘recovery rate ‘, ‘number of oocysts in straw samples’, ‘number of oocysts in calves’, ‘dry matter content of straw samples’ and ‘sporulation rate’ was significantly different from normal distribution. To determine the role of the different oocyst concentrations for the spiked straw samples on the median recovery rate, the Kruskal–Wallis test was used. To compare the four examined McMaster chambers for one tube, a Friedman test was performed. In both cases, a *p* value of < 0.05 was defined as significant. Spearman’s correlation coefficient (*ρ*_*Spearman*_) was calculated to investigate the associations between the mean OPG of faecal samples and the mean number of oocysts per gram of dry straw, the number of oocysts per gram of dry straw and moisture content of the sample. A *p* value of < 0.05 was defined as significant.

## Results

### Evaluation of the *Eimeria* oocyst recovery rate in straw spiked samples

**Fig. 2:**
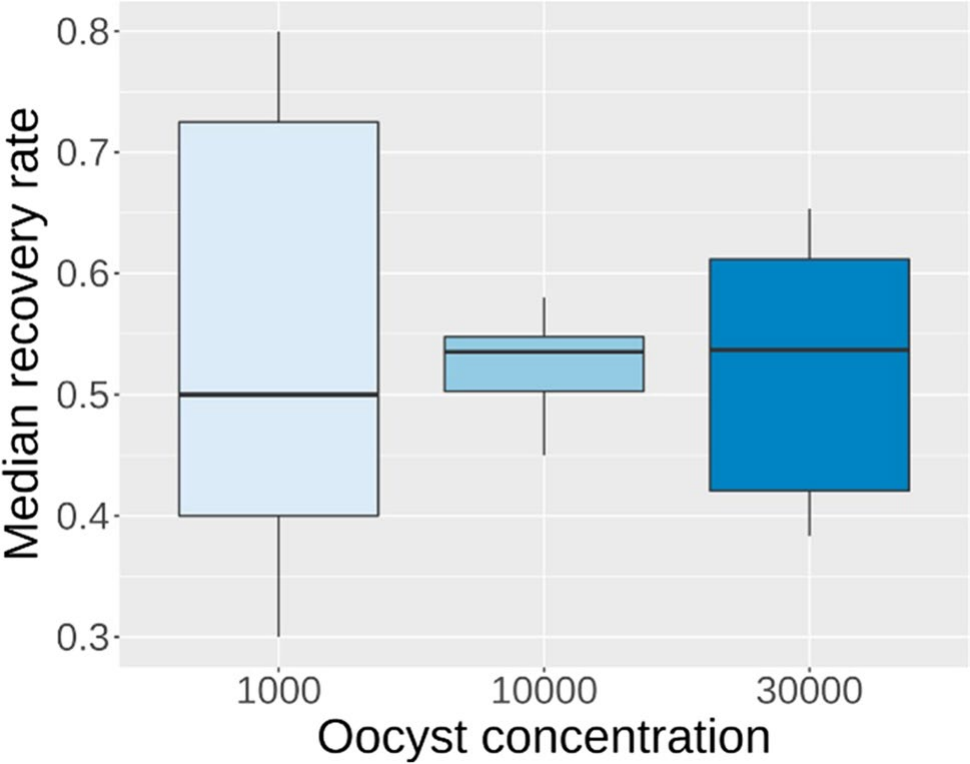
Median recovery rates of the spiked straw samples: 10 samples for each oocyst concentration (1000/10,000/30,000) were analysed

The mean recovery rate was 52.4% ± 11.1 SD (95% CI: 48.2–56.5%). The highest variability occurred in samples spiked with 1000 oocysts (*SD*_1000_ = 16.9%; *SD*_10,000_ = 3.3%; *SD*_30,000_ = 10.0%) (Fig. 2). No difference of the median recovery rate between the three spiking concentrations was detected (*p* = 0.704).

Comparing the numbers obtained from the four McMaster slides of each sample, a difference was evident only between the McMaster slides of samples spiked with 1000 oocysts (*p* = 0.007).

### Detection of Eimeria oocysts

Oocysts of the genus *Eimeria* were found on all four farms. Except for one examination day, on which all faecal and straw samples were negative (Table 1: farm 2, day 1), oocysts were detected in both faecal and straw samples.

**Table 1:** Quantitative *Eimeria* oocysts (OPG: oocysts per gram) detection in individual faecal calf and 12 environmental straw samples obtained on different examination days on four different farms. *SD* standard deviation, *CV* coefficient of variation

Farm no	1			2		3					4			
Month of collection	October			December		January					February			
Faecal samples														
Number of sampled animals	13			18			19				11			
Day of examination	21	27	47	1	26		5	7	14	28	1	5	7	14
*Eimeria* positive animals (*n*)	12	11	8	0	5		4	8	7	10	5	7	3	4
%	85.7	78.6	61.5	0.0	27.8		21.1	42.1	36.8	52.6	45.5	63.6	27.3	36.4
Calves with OPG > 5000 (*n*)	4	3	0	0	0		1	2	1	2	1	2	0	0
Maximum OPG	30950	37500	1650	0	900		29500	22700	5250	19400	71000	10350	1850	4100
Median OPG	1050	1100	100	0	0		0	0	0	150	0	150	0	0
Mean OPG	5432	4929	319	0	100		1574	1700	418	2005	6759	2059	323	609
SD OPG	8978.2	10179.5	475.0	0.0	230.1		6762.9	5369.3	1281.5	4699.7	21314.6	21314.6	642.4	1377.8
Straw samples (*n* = 12 per farm and examination day)														
Max OPG^a^	37692	8536	1792	0	1200		5284	410	673	2993	2108	5061	1542	317
Min OPG^a^	2339	1699	428	0	0		25	0	0	165	0	0	0	0
Median OPG^a^	12527	4674	1372	0	0		53	31	196	821	114	112	293	36
Mean OPG^a^	13712	4536	1218	0	167		518	64	277	1295	358	598	358	76
SD OPG^a^	9533.8	1837.3	468.3	0.0	360.1		1504.1	114.7	246.4	1097.0	623.9	1431.9	403.2	96.3
CV^a^	0.7	0.4	0.4	0.0	2.2		2.9	1.8	0.9	0.8	1.7	2.4	1.1	1.3
Sporulated^b^ (*n*)	8	12	12	0	0		0	0	1	4	0	0	0	0
Min sporulation rate (%)^c^	0	2	2	0	0		0	0	0	0	0	0	0	0
Max sporulation rate (%)^d^	14	40	12	0	0		0	0	2	16	0	0	0	0

^a^Per gram of dry straw

^b^Number of samples containing sporulated oocysts

^c^Minimum sporulation rate

^d^Maximum sporulation rate

#### Detection of Eimeria oocysts in field straw samples

**Fig. 3:**
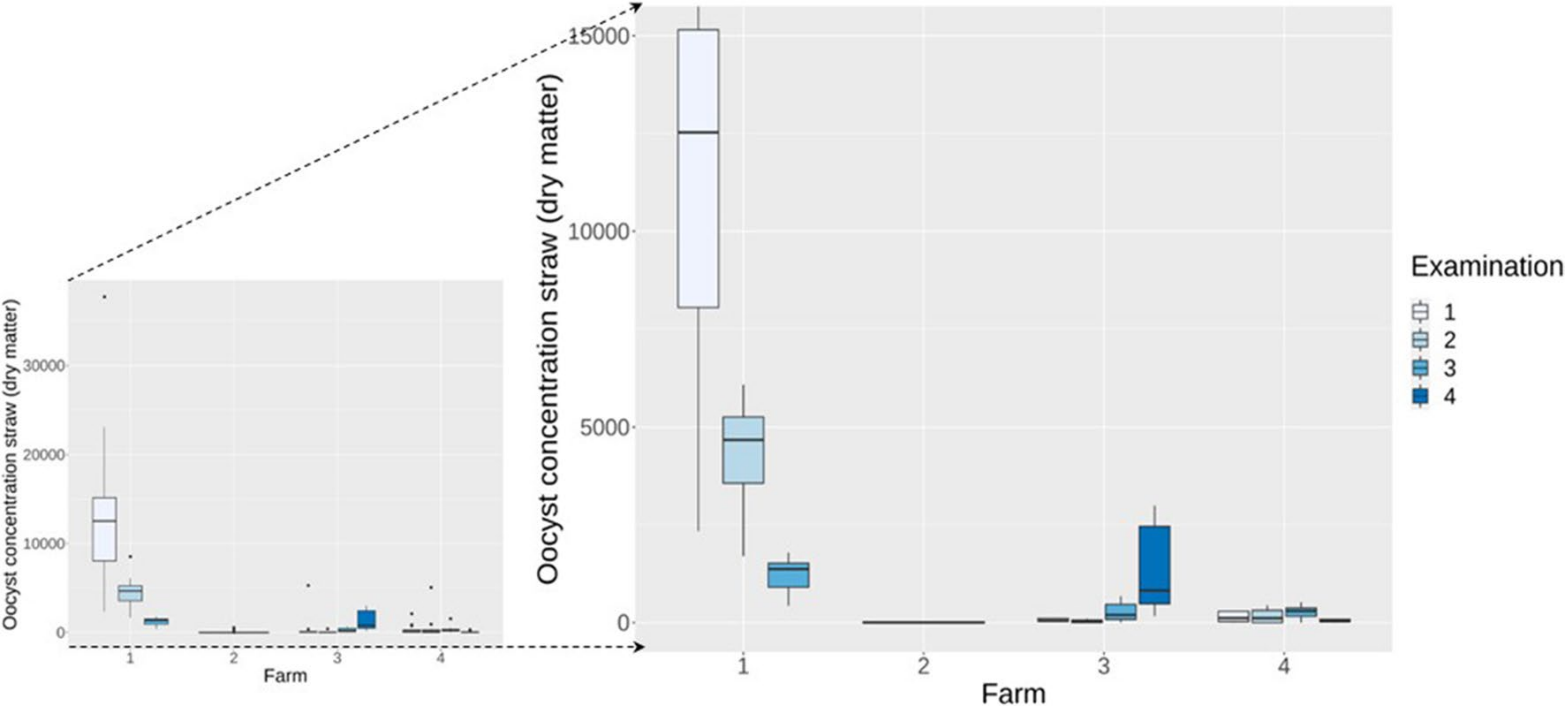
Boxplots with the number of oocysts per gram of dry straw samples (*n* = 156) on all examination days (2–4) on the four farms. Left side includes all data; on the right side, the outliers were eliminated, and the maximum oocyst number limited to 15,000

**Fig. 4:**
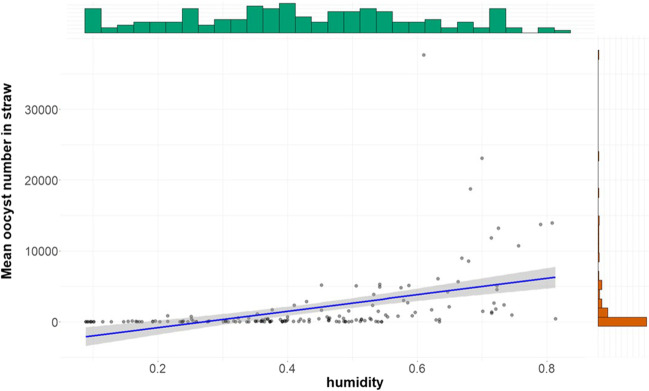
Positive correlation (*ρ*_*Spearman*_ = 0.7; *p* ≤ 0.005) between the mean number of *Eimeria* oocysts per gram of dry matter and the moisture content of the straw samples

Overall, 156 straw samples were examined. The number of oocysts in straw samples was highly variable between the farms and between the days of examination on a farm (Fig. 3). The number of oocysts per gram of dry matter was higher in samples with a higher moisture content (*ρ*_*Spearman*_ = 0.7; *p* ≤ 0.005; Fig. 4). Sporulated oocysts were observed only at farms 1 and 3 (Table 1). The overall sporulation rate was considered low: out of 156 samples, only 37 (23.7%) contained sporulated *Eimeria* oocysts, and the maximal sporulation rate was 40%.

#### Detection of Eimeria oocysts in faecal samples

Overall, 43.1% (84/195) of the faecal samples and 40 out of 61 (65.6%) calves were at least once positive for *Eimeria* oocysts. Clinical cases of coccidiosis occurred only on farm 1: four animals (two animals each on the first and second examination day) were treated with diclazuril (Vecoxan®, oral suspension, 1 mg/kg body weight).

**Fig. 5:**
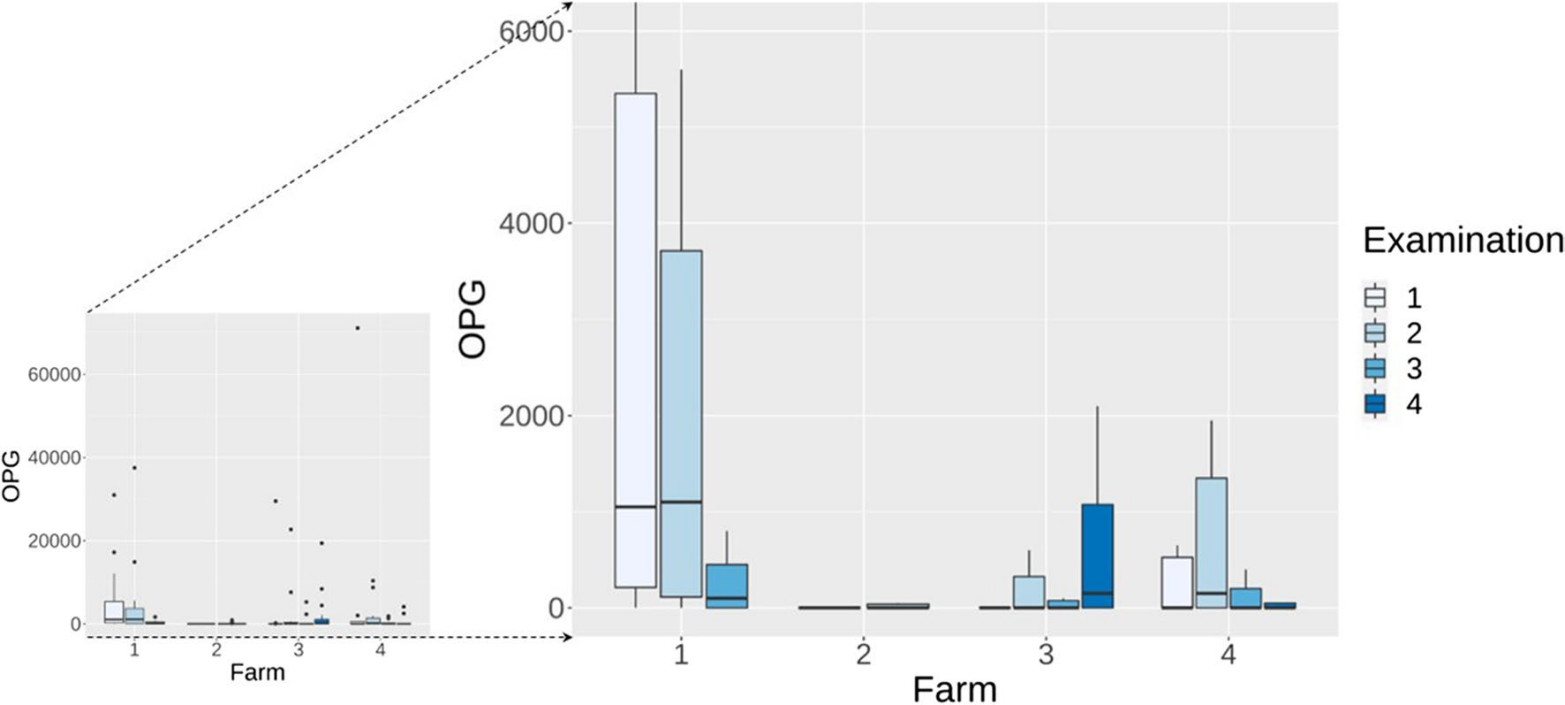
Boxplots with the number of oocysts per gram of faeces (OGP) of all calves (*n* = 61) on all examination days (2–4) on the four farms. Left side includes all data; on the right side, the outliers were eliminated and the maximum OPG value limited to 6000

There was high variation in the excretion of *Eimeria* oocysts between the farms and between the examination days (Fig. 5). Farm 1 had the highest number of positive calves (12/14), and these also had high OPG numbers (Table 1). In contrast, all animals on farm 2 were negative at the first examination day, followed by 5 positive animals at the second examination day, shedding a low number of oocysts. Similarly, farms 3 and 4 had a low number of positive animals with low OPGs, but single calves had high OPG numbers (Table 1).

#### Comparison between straw and faecal samples

**Fig. 6:**
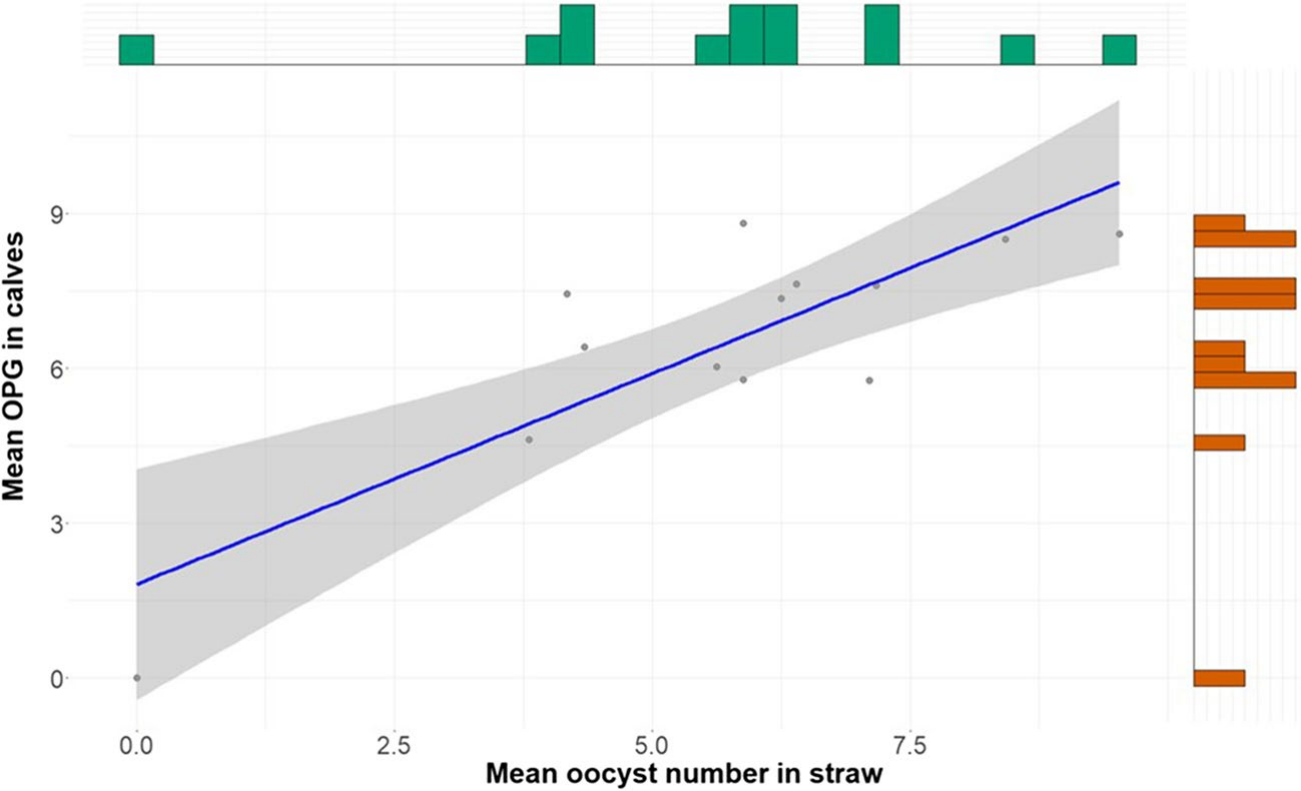
Positive correlation (*ρ*_*Spearman*_ = 0.60; *p* = 0.03) between the mean *Eimeria* OPG excreted by the calves and the mean number of oocysts per gram of dry matter in the straw samples on specific examination days

Across all farms, the mean value of the number of oocysts in the straw correlated positively with the mean value of the oocysts excreted in the faeces of all calves (*ρ*_*Spearman*_ = 0.60; *p* = 0.03; Fig. 6): the higher the OPG from the faeces, the higher the OPG values from the straw samples. The repeated examinations on the farms showed that the OPGs obtained from faeces and from straw samples fluctuated in a comparable way (Table 1; Fig. 3 and 5).

## Discussion

In the here presented study, we evaluated an inexpensive and easy to apply method for the isolation of *Eimeria* oocysts from environmental straw samples collected from calf barns. The goal of our study was to determine if the quantification of *Eimeria* oocysts from straw samples was comparable to the collection and analysis of individual faecal samples from calves kept in groups, with the aim to overcome dispendious and invasive individual rectal samplings for the determination of the infection dynamics within the group.

With *Eimeria* oocyst spiked straw samples, we obtained a mean recovery rate of 52.6%. This rate is high, compared to a previously described method to isolate *Eimeria* oocysts from soil samples, aiming to measure the contamination with *Eimeria* oocysts on pastures, with a recovery rate of 22% (Lassen and Lepik 2014). To our knowledge, there is no other comparable study for the detection of *Eimeria* oocysts performed on environmental barn straw samples. Furthermore, in our study, no correlation between numbers of oocysts added to the straw samples and the median recovery rates was observed. A difference was identified in the repeated counting of samples spiked with 1000 oocysts: due to this high variability, for samples with a low number of oocysts, it is suggested to count out more than one McMaster slide to obtain more accurate results.

Depending on the *Eimeria* species and many other variables, the animals may excrete *Eimeria* oocysts over a longer period. Together with the high tenacity of the oocysts in the environment, this could lead to an increasing number of oocysts in the straw over time. Although the hygienic measures as well as other management practices (e.g., frequency of straw replacement, amount of added straw) may affect the accumulation of oocysts in the environment, our repeated examinations in the different stables showed that there was no such accumulation in straw over time in the barns. Moreover, there was an overall low sporulation rate in the examined environmental straw samples. Most of the sampling took place in the colder season: lower temperatures limit the sporulation, and clinical cases are less frequent (Marquardt 1960; Daugschies and Najdrowski 2005). Accordingly, farm 1 sampled in October had the highest sporulation rates and was the only farm with clinical cases of eimeriosis. Overall, despite lack of standardisation of the management practices, our results confirm that daily fresh bedding can have a positive influence by limiting the number of oocysts in the environment, representing therefore a protective factor for coccidiosis (Lassen et al. 2009a, b; Bangoura et al. 2011). Also, the identified positive correlation between straw sample moisture and the number of oocysts per gram of dry matter additionally supports the relevance of fresh and dry bedding to reduce the pressure of infection with oocysts on calves.

The present study was a pilot study. Against this background, some limitations need to be considered. Four farms were investigated, showing high variability regarding *Eimeria* oocyst presence. This can be attributed to farm specific differences in management practises, animal husbandry and weather conditions (Bangoura and Bardsley 2020; Tomczuk et al. 2015; Cruvinel et al. 2021). Besides, the four study farms were visited and material collected repeatedly over 2–4 days throughout a sampling period of 47 days after arrival of calves at the farm. This may have additionally contributed to a certain level of variability of the data, as it has been shown that excretion dynamics of *Eimeria* spp. in calves vary in a time-dependent manner (Faber et al. 2002; Lopez-Osorio et al. 2020; Sánchez et al. 2008). However, our focus was on the development of a method for environmental sample collection and oocyst quantification. Therefore, the influence of the time point of sampling was considered rather minor. Several further factors that are potentially important for a risk analysis have not been fully considered in this study: (i) the specific animal density in the single barns, influencing oocyst accumulation and the potential risk of an outbreak (Tomczuk et al. 2015); (ii) the number of the collected straw samples was limited to 12, independently from the barn size; (iii) no differentiation between pathogenic and apathogenic *Eimeria* spp., which is relevant and could be carried out at the same time as the sporulation rate is analysed (Keeton and Navarre 2018) and, not least, (iv) climatic factors such as temperature and oxygen, influencing the exogenous sporulation of oocysts (Marquardt 1960; Marquardt et al. 1960; Duszynski and Conder 1977), were not evaluated. Knowledge on the microclimate in the specific barn (e.g. temperature and humidity) could be useful to identify the optimal conditions for oocyst sporulation (and therefore infectivity) in the specific environment (Schüller et al. 2013).

Yet, the standardisation of the collection and analysis of environmental straw samples presented in this study may exactly counteract the high variability of all mentioned conditions on farms and at individual levels and, instead, reflect in a normalised manner the evaluation of on-site *Eimeria* oocysts infection pressure. Therefore, the technique can be considered as a preliminary diagnostic approach estimating the risk for clinical coccidiosis in calves. Overall, the method showed a good recovery rate, was easy to perform, non-invasive, and material costs were low. Importantly, by comparing the *Eimeria* oocyst recovery data from straw with direct oocyst quantification from faeces of naturally infected calves in the same surrounding, we observed a significant positive correlation. With the examination of straw samples in stables, we cannot recognise whether a few animals excrete many oocysts, or if many animals excrete few oocysts. We also cannot exclude the occurrence of clinical coccidiosis despite low levels of contamination of the straw. Accordingly, the method described here is not a replacement for individual faecal samples, and if animals show clinical signs, individual diagnostic procedures must be applied. However, the method can be applied at group level, and, together with other risk factors mentioned above, may allow the assessment of the risk for clinical coccidiosis through sampling of environmental samples. It represents a less laborious sampling procedure promoting the well-being of young animals in a delicate phase of their life, by anticipating and supporting decisions related to preventive measures to be taken. This in turn may have the potential to reduce veterinary treatments and costs and propagate prevention in favour of reduced use of drugs.

## Data Availability

Detailed data not shown are available on request from the authors. The same applies to remaining material used for biomolecular analyses and, partially, frozen faecal samples.
